# Thermodynamic Analysis of Ti_3_O_5_Nanoparticles Formed in Melt and Their Effects on Ferritic Steel Microstructure

**DOI:** 10.3390/ma11081343

**Published:** 2018-08-02

**Authors:** Yanlin Wang, Meng Zhou, Xiaolu Pang, Xiaohua Chen, Zidong Wang, Alex A. Volinsky, Hao Tang

**Affiliations:** 1 School of Mechanical Engineering, Dongguan University of Technology, Dongguan 523808, China; Wangyanlin921@aliyun.com; 2 School of Materials Science and Engineering, University of Science and Technology Beijing, Beijing 100083, China; pangxl@mater.ustb.edu.cn (X.P.); mwtwfx@163.com (H.T.); 3 State Key Laboratory for Advanced Metals and Materials, University of Science and Technology Beijing, Beijing 100083, China; chenxh@skl.ustb.edu.cn; 4 Department of Mechanical Engineering, University of South Florida, Tampa, FL 33620, USA; volinsky@usf.edu

**Keywords:** metallurgy, nanoparticles, thermodynamics analysis, microstructure, ferritic steel

## Abstract

Based on the Wagner’s formalism combined with mass conservation, a thermodynamic analysis method has been developed previously. This method enables the calculation of the equilibrium matrix composition, precipitate composition and precipitate total molar fraction for Ti_*x*_O_*y*_(s) in molten metal, which can be determined at any appropriate temperature. In this present study, the Ti_3_O_5_ phase precipitation and the quantitative relationship between the addition of Ti, O and Ti_3_O_5_ in the molten steel were studied using the thermodynamic model. Using the combined multipoint dispersion supply method, electromagnetic stirring and well-dispersed 5-nm Ti_3_O_5_ nanoparticles were fabricated in the ferrite matrix of the as-cast high-strength steel with 0.05 wt % Ti—0.002 wt % O. The as-cast microstructure was improved by the homogeneously dispersed Ti_3_O_5_ nanoparticles through heterogeneous nucleation and grain refinement.

## 1. Introduction

The addition of microalloyed elements into steel always results in the formation of either solid solution or compounds, which produce different effects [1]. For instance, traces of titanium can significantly improve the comprehensive properties of the high-strength low-alloy (HSLA) steels through deoxidation, nitrogen fixation and desulphurization [2,3]. In terms of deoxidation, titanium always combines with oxygen to form different types of oxides, such as TiO, TiO_2_, Ti_2_O_3_ and Ti_3_O_5_. The oxide type proposed was mainly determined by the actual concentration of titanium [4]. By using the electron backscatter diffraction (EBSD), Cha et al. found that 0.001–0.2 wt % titanium induces the stable Ti_3_O_5_ phase although the stable phase is Ti_2_O_3_ when the titanium content reaches 0.2–2 wt % [5,6]. Furthermore, when the content of titanium is less than 0.4 wt % and 0.4–2 wt % in molten steel, the stable phases of Ti_3_O_5_ and Ti_2_O_3_ will be formed, respectively [7].

Improving the performance of products and saving resources are the main countermeasures for the iron and steel enterprises. In particular, the accurate design of material composition, organization and performance is the unremitting pursuit of material researchers. Therefore, the development of new steel materials has become a key research project in various countries [8,9]. In recent decades, a considerable number of studies focusing on the multivariable secondary phases and their applications in steel have been conducted by the material and metallurgical workers and great progress has been made in practice [10,11,12]. The effects of in-situ Ti_3_O_5_ nanoparticles on the mechanical properties of HSLA steels were studied experimentally in previous works [1,2] where the precipitation strengthening effect of Ti_3_O_5_ nanoparticles enhanced the steel mechanical properties. Conventionally, the corresponding compounds formed in molten steel are generally considered to be micron-sized particles and the nanoparticles-strengthened ferritic steels are typically referred to as oxide dispersion-strengthened (ODS) steels just through externally adding nano-oxide powders [11,13]. However, we found that a high density of in-situ homogeneously dispersed Ti_3_O_5_ nanoparticles can be obtained in the special steel melt containing Ti through scientifically designing the material chemical composition, which will be of great significance in refining the solidification microstructure of steel as well as improving macrosegregation, strength and toughness. Furthermore, the nanoparticles formed in melt so these particles have high melting points and are not easy to dissolve and grow in the subsequent hot-working process, which can improve the softening problem in the hot-working process and so on. Therefore, it is crucial to understand the formation mechanism of Ti_3_O_5_ phases in melt in order to engineer innovative HSLA steels with an optimal microstructure and excellent mechanical properties. 

Thermodynamic analysis is very important in the process of accurately designing materials. This is because the thermodynamic models enable the calculation of equilibrium composition in multivariable secondary phases, while the compositions of elements in the secondary phases as functions of content and temperature were developed based on solubility product formulas and mass conservation. This enables the calculation of equilibrium composition and relative amounts of multivariable secondary phases as a function of steel composition and temperature. The corresponding precipitation behavior of secondary phases was verified in the microalloyed steel systems, such as Ti-Nb-N-C, Ti-V-N-C, etc. [13,14]. At present, the formation mechanism of Ti_3_O_5_ nanoparticles in the Fe-Ti-O system molten steel has yet to be discovered. In this present study, based on the Wagner’s formalism and mass conservation combined with thermodynamic analysis, the precipitation behavior of Ti_3_O_5_ in molten steel was investigated, the effects of Ti, and O concentration on the formation of in-situ Ti_3_O_5_ nanoparticles were studied and their effects on the as-cast microstructure by the in-situ Ti_3_O_5_ nanoparticles in ferritic steel were also studied. Furthermore, we carried out microstructure characterization by scanning and transmission electron microscopy (SEM, TEM), high-resolution TEM (HRTEM) and atom probe tomography (APT) etc.

## 2. Experimental Procedure

### 2.1. Materials and Methods

A novel route was adopted to produce in-situ nanoparticles in molten metal as follows. First, the base materials (such as Si-Fe, Mn-Fe, Cr-Fe, Mo-Fe, V-Fe, Nb-Fe, Fe etc.) are placed in a vacuum melting furnace. After it is fully melted and the solidification temperature of molten steel is in the range of 1530–1620 °C, the pure Ti wire is added for multipoint dispersion supply. It is worth noting that electromagnetic stirring (about 4-KHz frequency) always exists during the whole melting process under the effect of electromagnetic stirring, while the flowing linear velocity of the molten metal is about 10–20 m s^−1^. Temperature is held constant for about 60 s after all Ti wires are completely melted. After this, the molten metal is cast into a crucible in air, the weight of ingot is about 8kg and its dimensions are135 mm × 75 mm × 96 mm. The flow linear velocity of the molten metal during the casting procedure is not less than 1.7 m s^−1^. The cooling rate is about 500 °C min^−1^ [15]. The chemical composition (in wt %) for the investigated high-strength steel used in this work is listed in Table 1, while the chemical composition of this steel was developed by University of Science and Technology Beijing together with Wuyang Iron & Steel technology Co., Ltd.

### 2.2. Thermodynamics Analysis

The microelements in steel mainly form solid solutions with the iron matrix or contribute to the formation of the corresponding compounds. For the Fe-Ti-O system, the thermodynamics calculation of the equilibrium matrix composition, precipitate composition and precipitate total molar fraction of Ti_*x*_O_*y*_(s) can be determined at any appropriate temperature based on the Wagner’s formalism combined with mass conservation. The Wagner’s equation has been widely used to express the activity coefficients of solutes in multi-component solutions.

(1) Wagner’s formalism

The chemical reaction between titanium and oxygen in liquid iron and its equilibrium constant are given by Equations (1) and (2) in the presence of solid Ti_*x*_O_*y*_:

(1) $${\mathrm{Ti}}_{x}O_{y}(s)=x\mathrm{Ti}+yO,$$

(2) $$K=(h_{\mathrm{Ti}}^{x}\cdot h_{O}^{y})/a_{{\mathrm{Ti}}_{x}O_{y}(s)}=\left\{f_{\mathrm{Ti}}^{x}{\left[\mathrm{Ti}\right]}^{x}\cdot f_{O}^{y}{\left[O\right]}^{y}\right\}/a_{{\mathrm{Ti}}_{x}O_{y}(s)},$$

where *K*, *a*, *h*, *f* and [i] denote the equilibrium constant, the Raoultian activity, the 1 wt % Henrian activity, the Henrian activity coefficient and concentration of *i* in liquid iron (wt %), respectively. The standard states of *h*_Ti_ and *h*_O_ in Equation (2) are infinite dilute solutions of titanium and oxygen in liquid iron. Taking the logarithm of both sides and rearranging Equation (2), we obtain the following:

(3) $$\mathrm{lg}K=x(\mathrm{lg}f_{\mathrm{Ti}})+y(\mathrm{lg}f_{O})+x(\mathrm{lg}[\mathrm{Ti}])+y([\mathrm{lg}[O])-\mathrm{lg}a_{{\mathrm{Ti}}_{x}O_{y}(s)}.$$

Each activity coefficient in Equation (3) can be expressed as:

(4) $$\mathrm{lg}f_{\mathrm{Ti}}=\sum_{i=1}^{n}e_{\mathrm{Ti}}^{i}[i]+\sum_{i=1}^{n}r_{\mathrm{Ti}}^{i}{\left[i\right]}^{2}+\sum_{i=1}^{n}r_{\mathrm{Ti}}^{i,\mathrm{Ti}}[i][\mathrm{Ti}],$$

(5) $$\mathrm{lg}f_{O}=\sum_{i=1}^{n}e_{O}^{i}[i]-\sum_{i=1}^{n}r_{O}^{i}{\left[i\right]}^{2}-\sum_{i=1}^{n}r_{O}^{i,O}[i][O].$$

Therefore, we can obtain the following:

(6) $$\begin{array}{l} \mathrm{lg}\left\{{\left[\mathrm{Ti}\right]}^{x}{\left[O\right]}^{y}\right\}=\mathrm{lg}K_{{\mathrm{Ti}}_{x}O_{y}}-\sum_{i=1}^{n}xe_{\mathrm{Ti}}^{i}[i]-\sum_{i=1}^{n}xr_{\mathrm{Ti}}^{i}{\left[i\right]}^{2}-\sum_{i=1}^{n}xr_{\mathrm{Ti}}^{i,\mathrm{Ti}}[i][\mathrm{Ti}]-\sum_{i=1}^{n}ye_{O}^{i}[i]-\\ \sum_{i=1}^{n}yr_{O}^{i}{\left[i\right]}^{2}-\sum_{i=1}^{n}yr_{O}^{i,O}[i][O]+\mathrm{lg}a_{Ti_{x}O_{y}(s)} \end{array}$$

where $e_{i}^{j}$, $r_{i}^{j}$, $r_{i}^{j,i}$ and [i] denote the first and second order interaction parameters between *i* and *j*, cross product term and concentration of *i* in liquid iron (wt %), respectively. It was assumed that the second order parameter and the cross product term could be ignored in the present work and $a_{{\mathrm{Ti}}_{x}O_{y}(s)}$ was unified because Ti*_x_*O*_y_*(s) was considered as a pure solid.

The main elements interaction activity coefficient of solid solubility of the product is listed in Table 2.

(2) Mass conservation

The addition of each microelement into steel must conform to the law of mass conservation. Therefore, the total composition of each microelement is constant in the formation of either a solid solution or compound. Based on the mass conservation of the species during the reaction, the following equations can be obtained:

(7) $$\frac{\mathrm{Ti}}{A_{\mathrm{Ti}}}-\frac{\left[\mathrm{Ti}\right]}{A_{\mathrm{Ti}}}=x\cdot t,$$

(8) $$\frac{O}{A_{O}}-\frac{\left[O\right]}{A_{O}}=y\cdot t,$$

where A_Ti_ and A_O_ are the atomic weights of titanium and oxygen, respectively. Ti and O are the mass fractions of titanium and oxygen elements, while *t* (mol) is the total molar fraction of the Ti*_x_*O*_y_*(s) formed in the liquid iron, respectively. Thus, the following Equation (9) can be acquired by combining Equations (7) and (8):

(9) $$\frac{\mathrm{Ti}}{A_{\mathrm{Ti}}}+\frac{O}{A_{O}}=\frac{\left[\mathrm{Ti}\right]}{A_{\mathrm{Ti}}}+\frac{\left[O\right]}{A_{O}}+(x+y)\cdot t.$$

The solid solution and precipitation behavior of Ti_3_O_5_ in the molten metal has been studied in the present work when the Ti*_x_*O*_y_*(s) is Ti_3_O_5_. Therefore, *x* = 3 and *y* = 5, A_Ti_ = 47.9 and A_O_ = 16. Furthermore, we know that:

(10) $$\mathrm{lg}K_{{\mathrm{Ti}}_{3}O_{5}}=-68280/T+19.95.$$

Accordingly, the thermodynamic model for the Ti_3_O_5_ can be described by Equations (6)–(10). There are three unknowns in Equations (6)–(10), which can be solved numerically to determine the equilibrium state. We know that for a given multicomponent steel Fe-Ti-O system at any appropriate temperature, the equilibrium matrix composition, precipitate composition and precipitate total molar fraction can be determined (i.e., [Ti], [O] and *t*). The numerical iteration calculation process was carried out in Matlab 8.1.

### 2.3. Tests

Microstructures were characterized using the Zeiss Supra 55 field emission scanning electron microscope (SEM), transmission electron microscopy (TEM), high-resolution TEM (HRTEM, FEI-Tecnai G^2^ 20) and atom probe tomography (APT). TEM samples were prepared by cutting thin wafers from the steel samples, which were mechanically thinned to ~35 μm. Three-millimeter discs were punched from the foils and electrochemically polished using a solution of 5% perchloric acid at −30°C, followed by ion-thinning to obtain an electron transparent area. The atomic level of particle characterization was performed by the Leap 3000 Hz atom probe tomography (APT) using a pulse repetition rate of 200 kHz and a 20% pulse fraction on the sample at 50 K. Atom probe tomography tip blanks (0.5 mm × 0.5 mm × 15 mm) were cut using electric discharge machining and were electropolished using a standard two-step procedure: 10% perchloric acid in acetic acid at 8−20 Vdc at room temperature, which was followed by 2% perchloric acid in butoxyethanol at 8−15 Vdc at room temperature.

## 3. Results and Discussion

### 3.1. Ti and O Effects on Precipitation Behaviour of Ti_3_O_5_

**Figure 1:**
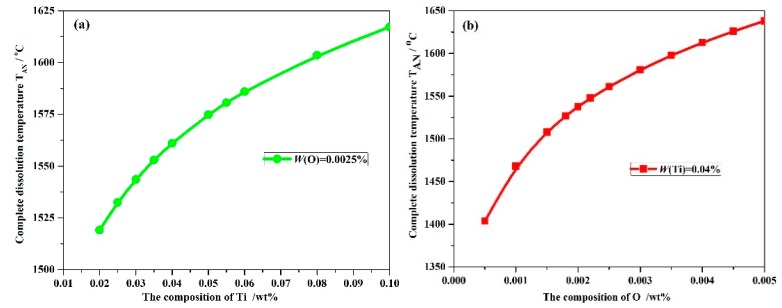
Complete dissolution temperature T_AN_, °C changes with: (**a**) O composition, wt % and (**b**) Ti composition, wt % in molten steels.

It is important to control the complete dissolution temperature T_AN_ of Ti_3_O_5_ in molten steels. However, little information is currently available regarding Ti_3_O_5_ in the molten metal. In this paper, according to Equations (6)–(10), the numerical iteration method was used to analyze the complete dissolution temperature for a series of Ti–O with (0.0005–0.005%)O and (0.02–0.1%)Ti composition. The complete dissolution temperatures for different Ti–O system steels are shown in Figure 1. The complete dissolution temperature of Ti_3_O_5_ increases with higher O or Ti levels in molten metal. It should be noted that the effect of O addition on the complete dissolution temperature is expected to be greater compared with Ti. If the complete dissolution temperature is below the liquidus temperature, the Ti_3_O_5_ compounds would not become precipitated in molten metal. Thus, it is important to optimize the concentration of Ti and O elements in the actual steel production.

**Figure 2:**
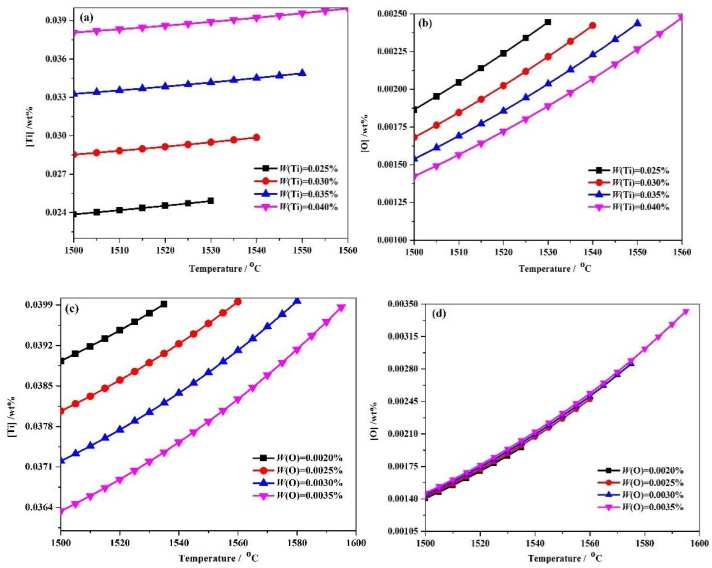
The evolution of (**a**,**c**) [Ti], wt % and (**b**,**d**) [O], wt % in molten steel as a function of steel composition and temperature(in °C) in different Ti-O system obtained by thermodynamic analysis.

Equations (6)–(14) and the numerical iteration method were combined to analyze the equilibrium solution thermodynamic state in steels with 0.025–0.04 wt % Ti and 0.002–0.0035wt % O composition in terms of [Ti] and [O] concentrations in solution and the total molar amount (*t*) of Ti_3_O_5_ at different temperatures. As shown in Figure 2, the dissolved Ti and O contents in solution decreased with decreasing temperature. With the content of Ti increasing at a given temperature, the dissolved Ti content increases but the dissolved O content decreases (Figure 2a,b). For the molten steel of Ti 0.04 wt %-O 0.0025 wt % system, the [Ti] is 0.03798892 wt %, [O] is 0.0013804 wt % and the total molar amount (*t*) of precipitated Ti_3_O_5_is 1.39949 × 10^−5^ mol at 1500 °C. In contrast, with an increase in O content at a given temperature, the dissolved Ti content decreases, while the dissolved O content slightly increases in Figure 2c,d. For the Ti0.04 wt %-O0.0035 wt % system, the [Ti] and [O] are 0.03633819 wt % and 0.00146141 wt %, respectively, while the total molar amount (*t*) of precipitated Ti_3_O_5_ is 2.54822 × 10^−5^ mol at 1500 °C. These thermodynamic results not only suggest that Ti_3_O_5_ is capable of being formed in the molten steel, but indicate that the amount (e.g., volume fraction, density or size scale) of Ti_3_O_5_ precipitation can be tuned by altering the addition of Ti and O, which can correspondingly affect the strengthening effect of Ti_3_O_5_ particles.

### 3.2. Precipitation Behaviour of Ti_3_O_5_Nanoparticles in High-Strength Steel with Ti 0.05 wt %-O 0.002 wt %

**Figure 3:**
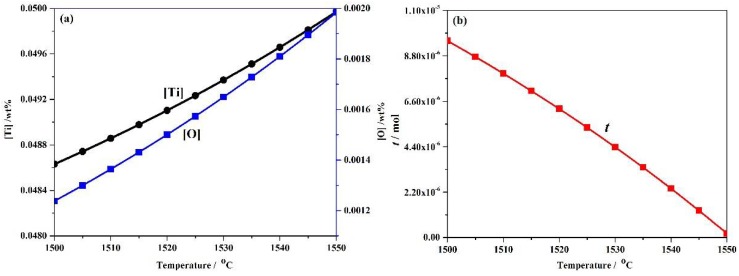
(**a**) [Ti], wt % and [O], wt % contents and (**b**) total molar amount, mol of Ti_3_O_5_ as a function of temperature(in °C) in Ti 0.05 wt %-O 0.0020 wt % system.

**Figure 4:**
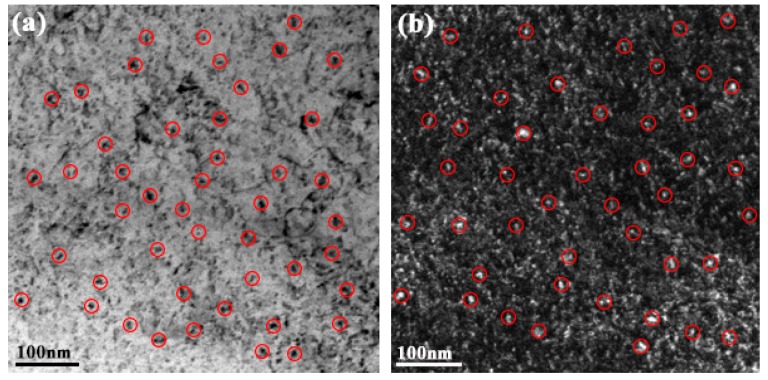
TEM images of the ferrite matrix in the as-cast high-strength steel of Ti 0.05 wt %-O 0.002 wt %: (**a**) Bright field; and (**b**) Dark field.

**Figure 5:**
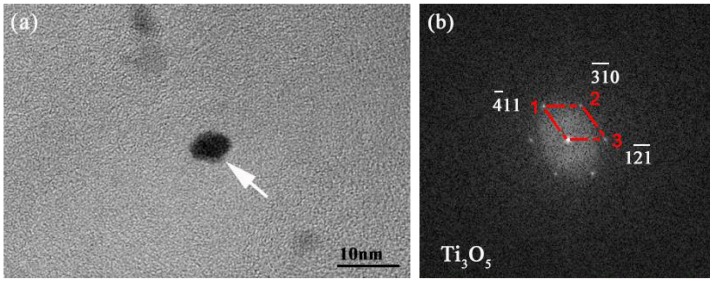
(**a**) HRTEM image of dispersed nanoparticles in high-strength steel; and (**b**) the corresponding FFT pattern of the black nanoparticles marked by white arrow in (**a**).

**Figure 6:**
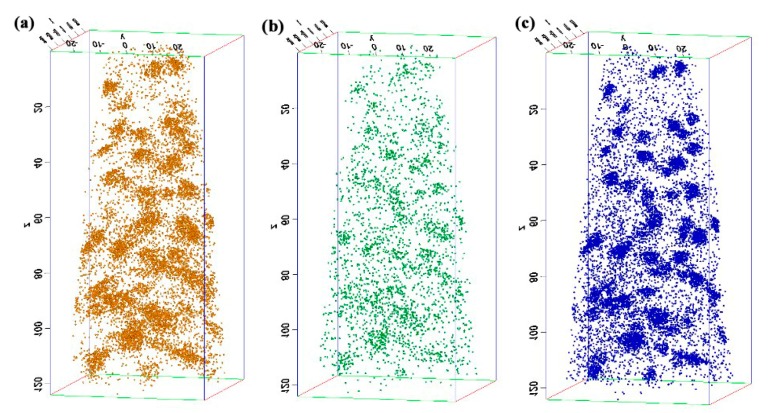
APT 3D atom reconstruction of high-strength steel samples showing distributions of various elements: (**a**) O; (**b**) Ti; and (**c**) Ti-O component.

The experimental high-strength steel with Ti 0.05 wt %-O 0.002 wt % was analyzed thermodynamically. The complete dissolution temperature T_AN_ in Ti 0.05 wt %-O 0.002 wt % system was 1555.21 °C, which was higher than the liquidus temperature of 1512.75 °C by 42.46 °C. This implies that Ti_3_O_5_ compounds can be precipitated in this molten steel. The thermodynamic results indicate that [Ti] and [O] contents both decreased in Figure 3a and the total molar amount (*t*) of Ti_3_O_5_ increased in Figure 3b with decreasing temperature. This means that the formation of high density Ti_3_O_5_ particles in the molten metal is feasible during the cooling process (e.g., casting in the crucible in air in this paper). At 1510 °C, the [Ti] and [O] total molar amount (*t*) and the volume fraction of Ti_3_O_5_ are 0.04886010 wt %, 0.00136540 wt %, 7.932 × 10^−6^ mol and 0.00325738 vol %, respectively. A pure Ti wire was added through multipoint dispersion supply and a strong convection stirring was applied during the melting process so the Ti atoms can be homogenized in the molten metal. Consequently, we anticipated that high density nanoscale Ti_3_O_5_ particles will form in the molten metal due to the uniform distribution and lower volume fraction (only 0.00325738 vol % at 1510 °C) of Ti atoms. Furthermore, the forced intense convection from the electromagnetic stirring can inhibit the growth of the Ti_3_O_5_ nanoparticles during solidification so many of the nanoparticles can remain small [23,24]. As a result, a high density of in-situ dispersed Ti_3_O_5_ nanoparticles can be obtained in the high-strength steel. This was confirmed by the TEM results in Figure 4 and Figure 5. In Figure 4, high density nanoparticles with the size of about 5 nm were homogeneously distributed in the ferrite matrix. The HRTEM image and FFT pattern indexing in Figure 5 indicate that the dispersed nanoparticles are the Ti_3_O_5_ phases. The calculated inter-planar spacing in real space corresponding to spots 1, 2 and 3 in the reciprocal space were 0.2234, 0.2567 and 0.2539 nm, coinciding with $\left(\bar{4}11\right)$, $\left(\bar{31}0\right)$ and $\left(1\bar{21}\right)$ lattice planes of Ti_3_O_5_, respectively [25]. Through TEM and HRTEM identification of the precipitated phase, the dispersed nanoparticles are determined to be Ti_3_O_5_. By combining the results of the APT experiment, this conclusion is further supported. Figure 6 shows the APT 3D atom reconstruction of high-strength steel samples showing distributions of various elements. Ti_3_O_5_contains three titanium atoms for every five oxygen atoms. Therefore, there are more oxygen atoms than titanium atoms in the elemental distribution.

**Figure 7:**
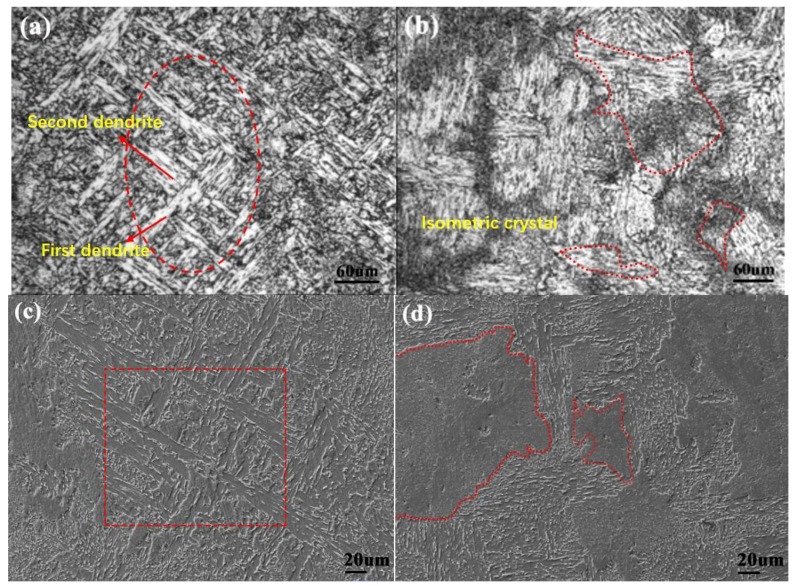
(**a**,**c**) The as-cast microstructure without dispersed in-situ nanoparticles; and (**b**,**d**) The as-cast microstructure with dispersed in-situ nanoparticles in high-strength steel.

Figure 7a,c show the typical dendritic structure of the as-cast steel without dispersed nanoparticles. Figure 7b,d show the isometric crystal microstructure of the as-cast steel with dispersed nanoparticles and elemental content of Ti 0.05 wt %-O 0.002 wt %. It is known that once Ti_3_O_5_ nanoparticles form in situ in molten steel, the as-cast microstructure and properties will be improved. This is due to heterogeneous nucleation and grain refinement caused by Ti_3_O_5_ nanoparticles. In addition, the in-situ nanoparticles formed in molten metal can act as the heterogeneous nucleation cores of the carbonitride and sulfide, resulting in uniform distribution, refinement of non-metallic inclusions and grain refinement in steels. 

## 4. Conclusions

Ti_3_O_5_phases can thermodynamically favor precipitation in molten steel. The amount of Ti_3_O_5_ phase formed in molten steel is correlated with the addition of Ti and O according to the quantitative thermodynamics study. TEM characterization shows that a high density of Ti_3_O_5_ nanoparticles with a size of about 5 nm are homogeneously dispersed in the ferrite matrix of as-cast high-strength steel with Ti 0.05 wt %-O 0.002 wt %. The as-cast microstructure was improved through heterogeneous nucleation by in-situ homogeneously dispersed Ti_3_O_5_ nanoparticles. The uniform nano-scale distribution of Ti_3_O_5_ precipitation can be attributed to the multipoint dispersion supply method of pure Ti and strong convection stirring from the electromagnetic stirring technique.

## Figures and Tables

**Table 1:** Chemical composition of high-strength steel (wt %).

C	Si	Mn	P ≤ 0.01	S ≤ 0.002	Cr	Mo	V	Nb	Ti	O	N
0.04	0.03	0.85	0.008	0.0016	0.45	0.5	0.03	0.06	0.05	0.002	0.0013

**Table 2:** Selected interaction activity coefficient in dilute solutions of steel.

Element *j*	$e_{\mathrm{Ti}}^{j}$	$e_{O}^{j}$
C	−221/*T* − 0.072 [16]	−0.42 [16]
Si	177.5/*T* − 0.12 [17]	−0.066 [16]
Mn	−0.043 [16]	−0.021 [16]
Cr	−0.016 [18]	−0.046 [18]
S	−0.27[16]	−0.133 [19]
P	—	—
Ti	212/*T* − 0.0640 [5]	−701/*T* + 0.0344 [5]
V	28.416/*T* + 0.0032 [20,21]	−2500/*T* + 1.01 [21]
Nb	15.74/*T* − 0.00314 [20,21]	−3440/*T* + 1.717 [21]
Mo	—	—
N	−19,500/*T* + 8.37 [22]	0.057 [19]
O	−2098/*T* + 0.0943 [6]	−1750/*T* + 0.760 [5]

— Values not found in the literature are assumed to be zero in current calculation.
